# Reply to Roesler, R.; Isolan, G.R. Comment on “Santana-Bejarano et al. NRP1 and GFAP Expression in the Medulloblastoma Microenvironment: Implications for Angiogenesis and Tumor Progression. *Cancers* 2025, *17*, 2417”

**DOI:** 10.3390/cancers18060931

**Published:** 2026-03-13

**Authors:** Marisol Godínez-Rubí, Margarita Belem Santana-Bejarano, María Paulina Reyes-Mata, José de Jesús Guerrero-García, Daniel Ortuño-Sahagún

**Affiliations:** 1 Laboratorio de Patología Diagnóstica e Inmunohistoquímica, Centro de Investigación y Diagnóstico en Patología, Departamento de Microbiología y Patología, Centro Universitario de Ciencias de la Salud (CUCS), Universidad de Guadalajara, Guadalajara 44340, Jalisco, Mexico; 2 Doctorado en Ciencias en Biología Molecular en Medicina, Centro Universitario de Ciencias de la Salud (CUCS), Universidad de Guadalajara, Guadalajara 44340, Jalisco, Mexico; 3 Departamento de Disciplinas Filosófico, Metodológicas e Instrumentales, Centro Universitario de Ciencias de la Salud (CUCS), Universidad de Guadalajara, Guadalajara 44340, Jalisco, Mexico; paulina.reyes@academicos.udg.mx; 4 Banco de Sangre Central, Unidad Médica de Alta Especialidad (UMAE), Hospital de Especialidades (HE), Centro Médico Nacional de Occidente (CMNO), Instituto Mexicano del Seguro Social (IMSS), Guadalajara 44340, Jalisco, Mexico; 5 Departamento de Farmacobiología, Centro Universitario de Ciencias Exactas e Ingenierías (CUCEI), Universidad de Guadalajara, Guadalajara 44340, Jalisco, Mexico; 6 Laboratorio de Neuroinmunobiología Molecular, Instituto de Neurociencias Translacionales, Centro Universitario de Ciencias de la Salud (CUCS), Universidad de Guadalajara, Guadalajara 44340, Jalisco, Mexico

We sincerely thank Roesler and Isolan [1] for carefully reading our manuscript and providing insightful, constructive feedback. We greatly value this type of scholarly exchange, as it demonstrates how integrating different experimental approaches can refine biological interpretation and contribute meaningfully to progress in medulloblastoma research.

We agree that biological inferences derived from mRNA expression data do not always align with those obtained from protein-level analyses. This divergence is well recognized and reflects the multiple regulatory layers governing gene expression, including alternative splicing, post-transcriptional regulation, post-translational modifications, and context-dependent protein interactions [2,3,4].

A main strength of our work is its focus on protein expression at the cellular level within the tumor microenvironment. Rather than relying on bulk tumor analyses, we specifically sought to determine which cell populations express NRP1 and to examine this expression within its spatial and structural context [5]. Using immunohistochemistry with a thoroughly validated antibody (anti-NRP1, clone EPR3113; ab81321), we detected the ~103 kDa transmembrane isoform of Neuropilin-1 (UniProt: O14786) [6], corresponding to the functionally active receptor. Importantly, our quantitative analysis was based on the density of NRP1-positive cells rather than total protein abundance, a parameter that captures a biological dimension distinct from transcriptomic measurements. This distinction is particularly relevant given our observation that most NRP1-positive cells in our cohort were tumor-associated macrophages and endothelial cells. Accordingly, we interpret our findings primarily within the framework of tumor microenvironment dynamics, immune regulation, and angiogenic remodeling, rather than as a direct surrogate of tumor cell–intrinsic gene expression. This interpretation is consistent with prior studies linking membrane-bound, functional NRP1 expression to angiogenesis, tumor progression, metastasis, and adverse clinical outcomes across multiple cancer types (an extensive and recent review of the topic is found in [7]).

From a mechanistic perspective, NRP1 regulation introduces an additional layer of complexity. The NRP1 gene, located at 10p11.22, produces at least 14 splice variants, generating isoforms ranging from approximately 60 to 923 amino acids [8,9]. Shorter variants typically encode soluble forms (sNRP1), while longer transcripts produce the transmembrane receptor. These isoforms may have distinct, and sometimes opposing, biological effects. In particular, soluble NRP1 acts as a decoy receptor by sequestering VEGF165 and limiting pro-angiogenic signaling [9], whereas the membrane-bound form promotes angiogenesis [7]. Such isoform-specific differences are not captured by bulk mRNA expression analyses.

NRP1 mRNA is also regulated by microRNAs, including miR-141 and miR-320, which have been shown to downregulate its expression in several cancer models, such as malignant melanoma [10], pancreatic [11], and oral carcinomas [12]. Thus, transcript-level measurements may already reflect upstream repressive mechanisms that are not necessarily mirrored at the protein level. Post-translational modifications, including glycosylation [13], further contribute to the structural and functional diversity of NRP1 protein species.

Overall, the differences observed between transcriptomic associations and our cell-resolved, protein-based findings do not represent true biological inconsistencies. Rather, they reflect distinct regulatory layers of NRP1 biology. From this perspective, transcriptomic and histopathological approaches should be considered complementary, each providing essential and nonredundant insights into the role of NRP1 in medulloblastoma.

We fully share Roesler and Isolan’s view that future studies integrating transcriptomics, proteomics, and spatially resolved methodologies will be critical for clarifying the prognostic and functional relevance of NRP1. In particular, collaborative efforts that combine large-scale gene expression datasets with detailed, cell-specific, and microenvironment-focused protein analyses may provide valuable opportunities to further elucidate the context-dependent roles of NRP1 across medulloblastoma subgroups.

We thank the authors again for their contribution to this discussion and for fostering a rigorous and constructive scientific exchange.

## References

[1] Roesler R., Isolan G.R. (2026). Comment on Santana-Bejarano et al. NRP1 and GFAP Expression in the Medulloblastoma Microenvironment: Implications for Angiogenesis and Tumor Progression. *Cancers* 2025, *17*, 2417. Cancers.

[2] Buccitelli C., Selbach M. (2020). mRNAs, Proteins and the Emerging Principles of Gene Expression Control. Nat. Rev. Genet..

[3] Saha A., Kim Y., Gewirtz A.D.H., Jo B., Gao C., McDowell I.C., Engelhardt B.E., Battle A., The GTEx Consortium (2017). Co-Expression Networks Reveal the Tissue-Specific Regulation of Transcription and Splicing. Genome Res..

[4] De Araújo M.A., Malafaia O., Ribas Filho J.M., Fratini L., Roesler R., Isolan G.R. (2023). Low Expression of the NRP1 Gene Is Associated with Shorter Overall Survival in Patients with Sonic Hedgehog and Group 3 Medulloblastoma. Int. J. Mol. Sci..

[5] Santana-Bejarano M.B., Reyes-Mata M.P., Guerrero-García J.D.J., Ortuño-Sahagún D., Godínez-Rubí M. (2025). NRP1 and GFAP Expression in the Medulloblastoma Microenvironment: Implications for Angiogenesis and Tumor Progression. Cancers.

[6] Bateman A., Martin M.-J., Orchard S., Magrane M., Adesina A., Ahmad S., Bowler-Barnett E.H., Bye-A-Jee H., Carpentier D., The UniProt Consortium (2025). UniProt: The Universal Protein Knowledgebase in 2025. Nucleic Acids Res..

[7] Varanasi S.M., Gulani Y., Rachamala H.K., Mukhopadhyay D., Angom R.S. (2025). Neuropilin-1: A Multifaceted Target for Cancer Therapy. Curr. Oncol..

[8] National Center for Biotechnology Information (NCBI) Gene ID: 8829. https://www.ncbi.nlm.nih.gov/gene?Db=gene&Cmd=DetailsSearch&Term=8829.

[9] Gagnon M.L., Bielenberg D.R., Gechtman Z., Miao H.Q., Takashima S., Soker S., Klagsbrun M. (2000). Identification of a Natural Soluble Neuropilin-1 That Binds Vascular Endothelial Growth Factor: In Vivo Expression and Antitumor Activity. Proc. Natl. Acad. Sci. USA.

[10] Graziani G., Lacal P.M. (2015). Neuropilin-1 as Therapeutic Target for Malignant Melanoma. Front. Oncol..

[11] Ma L., Zhai B., Zhu H., Li W., Jiang W., Lei L., Zhang S., Qiao H., Jiang X., Sun X. (2019). The miR-141/Neuropilin-1 Axis Is Associated with the Clinicopathology and Contributes to the Growth and Metastasis of Pancreatic Cancer. Cancer Cell Int..

[12] Wu Y.-Y., Chen Y.-L., Jao Y.-C., Hsieh I.-S., Chang K.-C., Hong T.-M. (2014). miR-320 Regulates Tumor Angiogenesis Driven by Vascular Endothelial Cells in Oral Cancer by Silencing Neuropilin 1. Angiogenesis.

[13] Huang X., Ye Q., Chen M., Li A., Mi W., Fang Y., Zaytseva Y.Y., O’Connor K.L., Vander Kooi C.W., Liu S. (2019). N-Glycosylation-Defective Splice Variants of Neuropilin-1 Promote Metastasis by Activating Endosomal Signals. Nat. Commun..

